# Predicting Mortality of Incident Dialysis Patients in Taiwan - A Longitudinal Population-Based Study

**DOI:** 10.1371/journal.pone.0061930

**Published:** 2013-04-23

**Authors:** Ping-Hsun Wu, Yi-Ting Lin, Tzu-Chi Lee, Ming-Yen Lin, Mei-Chuan Kuo, Yi-Wen Chiu, Shang-Jyh Hwang, Hung-Chun Chen

**Affiliations:** 1 Division of Nephrology, Department of Internal Medicine, Kaohsiung Medical University Hospital, Kaohsiung, Taiwan; 2 Department of Family Medicine, Kaohsiung Medical University Hospital, Kaohsiung, Taiwan; 3 Department of Public Health, College of Medicine, Kaohsiung Medical University, Kaohsiung, Taiwan; 4 Department of Internal Medicine, College of Medicine, Kaohsiung Medical University, Kaohsiung, Taiwan; 5 Faculty of Renal Care, College of Medicine, Kaohsiung Medical University, Kaohsiung, Taiwan; University of Louisville, United States of America

## Abstract

**Background:**

Comorbid conditions are highly prevalent among patients with end-stage renal disease (ESRD) and index score is a predictor of mortality in dialysis patients. The aim of this study is to perform a population-based cohort study to investigate the survival rate by age and Charlson comorbidity index (CCI) in incident dialysis patients.

**Methods:**

Using the catastrophic illness registration of the Taiwan National Health Insurance Research Database for all patients from 1 January 1998 to 31 December 2008, individuals newly diagnosed with ESRD and receiving dialysis for more than 90 days were eligible for our study. Individuals younger than 18 years or renal transplantation patients either before or after dialysis were excluded. We calculated the CCI, age-weighted CCI by Deyo-Charlson method according to ICD-9 code and categorized CCI into six groups as index scores <3, 4–6, 7–9, 10–12, 13–15, >15. Cox regression models were used to analyze the association between age, CCI and survival, and the risk markers of survival.

**Results:**

There were 79,645 incident dialysis patients, whose mean age (± SD) was 60.96 (±13.92) years; 51.43% of patients were women and 51.2% were diabetic. In cox proportional hazard models and stratifying by age, older patients had significantly higher mortality than younger patients. The mortality risk was higher in persons with higher CCI as compared with low CCI. Mortality increased steadily with higher age or comorbidity both for unadjusted and for adjusted models. For all age groups, mortality rates increased in different CCI groups with the highest rates occurring in the oldest age groups.

**Conclusions:**

Age and CCI are both strong predictors of survival in Taiwan. The older age or higher comorbidity index in incident dialysis patient is associated with lower long-term survival rates. These population-based estimates may assist clinicians who make decisions when patients need long-term dialysis.

## Introduction

End-stage renal disease (ESRD) patients have a high prevalence of comorbid conditions [1], [2], high mortality rate [3]–[5], and poor prognosis. Although the prognosis for patients with ESRD treated by dialysis has improved in recent years, mortality rates remain high. The ESRD population is high in Taiwan and patients start dialysis with very low residual renal function and in poor clinical conditions [6]. Compared with populations in western countries, cardiovascular disease occurs less frequently among Asians [7] and late dialysis in Taiwan with low mortality [6] is quite different from other countries. Besides, there is no difference in survival rates between hemodialysis and peritoneal dialysis patients in Taiwan [8], [9]. The median serum creatinine and glomerular filtration rate (GFR) are 10.1 mg/dL and 4.7 mL/min/1.73m^2^ in Taiwan and lower GFR at dialysis initiation is associated with lower mortality [10]. This finding is similar to the result in an IDEAL (Initiating Dialysis Early and Late) study that mentioned early initiation of dialysis principle was not associated with an improvement in survival or clinical outcomes [11]. Although late dialysis strategy is applied in Taiwan, comorbidity is still a major confounder but also a predictor of the patient’s natural course and outcomes. Therefore, comorbidity should be assessed in dialysis patients and simplified comorbidity indexes are more applicable. Comorbidity scales have been evaluated in dialysis patients, such as Charlson comorbidity index (CCI) [12], index of co-existent diseases (ICED) [13], Davies [14], and Wright-Khan indices [3]. Of the comorbidity scales developed for general medical patients, CCI is the most popular [12] and easiest to apply. CCI was originally developed to create a single-value summary for several comorbid conditions for breast cancer patients in 1984 and is suitable for general medical inpatient populations. It is also applied to the dialysis population and also validated in ESRD patients [5], [15]. ICED works better than CCI in analyses of ESRD patients but ICED is difficult to apply [13]. CCI is also a better predictor for mortality compared with the Davies comorbidity index for peritoneal dialysis patients [16]. However, previous studies examining on incident dialysis populations were performed in single centers, involved small numbers of patients, and had short-term duration. Few reports predict long-term mortality in dialysis patients [17], [18] and publications on this subject in Asian populations are rare [19]. Age with CCI as a good predictor of long term prognosis in dialysis patients in Taiwan is still unknown. The objective of the present study is to predict long term survival in large sample incident dialysis patients using CCI and age-weight CCI in a 10-year nationwide cohort study. We used claims data from the National Health Insurance program in Taiwan to evaluate the comorbidities during pre-dialysis care and to investigate the relationship between CCI and survival in incident patients. Population-based data often contains all patients with a given disease, and administrative data offer a picture of the “real world” effectiveness of interventions as they are being practiced.

## Methods

### Data Source

This study is based on a longitudinal health insurance database, the National Health Insurance Research Database (NHIRD), provided by the Taiwan National Health Research Institute. Taiwan launched its compulsory social insurance program, National Health Insurance (NHI), to provide health care for all the island’s residents since 1995. The annual coverage rate of the NHI program ranged from 96.16% to 99.6% and includes contracts with 97% of hospitals and clinics, with more than 23 million Taiwanese residents enrolled since 1997. It covers all medical benefit claims of ambulant and inpatient care and is extensively applied to many epidemiological studies. The NHIRD established a registry system for “Catastrophic Illnesses”, including cancer, chronic mental illness, end-stage renal disease, congenital illness, and several autoimmune diseases. Insured persons with major diseases can apply for catastrophic illness registration cards from the Bureau of National Health Insurance (BNHI) and do not need to make co-payments when seeking health care for catastrophic illness. Both outpatient and inpatient claims of beneficiaries with a catastrophic illness certificate are collected in the catastrophic illness profile and are distributed as a package. The BNHI performs routine validations of the diagnoses by reviewing the original medical charts of all of the patients who apply for catastrophic illness registration. In this study, all cases of dialysis patients are obtained from the Registry of Catastrophic Illness Database, a subpart of the NHIRD. The issuance of catastrophic illness certificates is validated by at least 2 specialists, based on careful examination of the medical records, laboratory studies, imaging studies, and dialysis treatment. Only individuals who meet the diagnostic criteria for major diseases are issued a catastrophic illness certificate. The database included all relevant information about the “catastrophic illness certificate” status, such as diagnostic codes in the format of the *International Classification of Disease, Ninth Revision, Clinical Modification* (ICD-9-CM), date of diagnosis, date of death, date of receiving dialysis, date of every clinic visits, details of prescriptions, expenditure amounts, and outpatient/inpatient claimed data for the beneficiaries with catastrophic illnesses during the period 1998–2008. During the study period, International Classification of Diseases, Ninth Revision (ICD-9) codes are used to define diseases. Personal information including family history, lifestyle, and habits such as smoking and alcohol use are not available from the NHIRD.

### Study Cohorts

From the Registry for Catastrophic Illness Patient Database, we selected all patients diagnosed ESRD defined as those who had catastrophic illness registration cards for ESRD (ICD-9-CM code 585) and started hemodialysis or peritoneal dialysis of more than 90 days of renal replacement therapy between Jan,1, 1998, and Dec, 31, 2008. The NHIRD are enrolled since 1997, so the study cohort started from 1998, as a wash-out period for one year, and we also extended the observation time until 2009 in this cohort study. We excluded individuals younger than 18 years (n = 377) or those who had renal transplantation either before or after dialysis (n = 1585). Patients receiving regular dialysis without catastrophic illness certificate were not included in this study.

Under the reimbursement system, hospitals have to claim medical expenses from the NHI based on the diagnosis or treatment codes for the disease presented by their patients. We used all diagnosis codes for a full year to define the existing comorbidities, including outpatient and inpatient diagnosis codes before the date of starting dialysis, which is defined as the index date. CCI and age-weighted CCI are calculated according to all diagnosis codes for a full year before the index date on every inpatient or outpatient to define the existing comorbidities [20]. Follow-up began on the index date until death or remaining alive at the end of the study period (Dec, 31, 2009). The CCI is defined by Charlson et al [12] and the Deyo-Charlson comorbidity index ( table S1), based on ICD-9 codes in claims data, has been widely used in the analyses of the impact of comorbidities on mortality [21]. The CCI contains the following components: 1 point is assigned for history of myocardial infarction, congestive heart failure, peripheral vascular disease, cerebrovascular disease, dementia, chronic pulmonary disease, connective tissue disorder, peptic ulcer disease, mild liver disease and diabetes without end organ damage; 2 points for hemiplegia, moderate to severe renal disease (excluded in our scale because all patients had diagnosis of ESRD) diabetes with end organ damage, tumor without metastases, leukemia, lymphoma and myeloma; 3 points for moderate or severe liver disease; and 6 points for metastatic solid tumor or full-blown acquired immunodeficiency syndrome (still included in this study’s scale because of small numbers of dialysis patients) (table S2). As for Age-weighted-CCI, 1 point is added to the score for every decade more than 40 years of age (0 points for 18–49 years, 1 point for 50–59 years, 2 points for 60–69 years, 3 points for 70–79 years, 4 points for 80–89 years, 5 points for 90–99 years) (table S3). Because all patients are on dialysis, the minimum Charlson score is 2. All dialysis patients with diabetes are defined as diabetes with end organ damage. We categorized CCI into six groups as index scores ≦3, 4–6, 7–9, 10–12, 13–15, >15. Hazard ratio of mortality in dialysis patients by six comorbidity index groups and different age groups were analyzed.

### Statistical Analysis

Data is summarized using proportions, and means (±standard deviation) as appropriate. The association between CCI and mortality was assessed using Cox proportional hazards model and Kaplan–Meier estimate with log rank tests declaring survival in the follow-up period for dialysis patients for each CCI level. In the mortality analyses, the patients were followed until event (death) or censoring (lost to follow-up or end of follow-up period); which ever happened first. The variables were analyzed initially by univariate analysis, and statistically significant variables were chosen for multivariate analysis. Analyses were performed using the SAS statistical package (version 9.2; SAS Institute Inc, www.sas.com). All statistical tests were 2 sided. *p*-value <0.05 was considered statistically significant.

## Results

**Figure 1:**
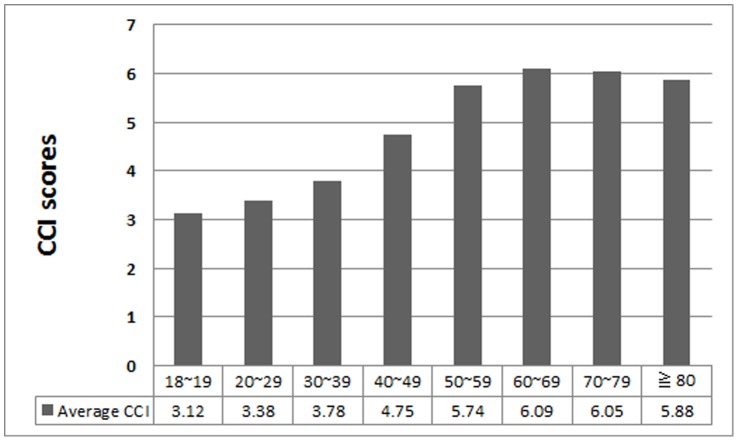
Average comorbidity scores by age group.

**Figure 2:**
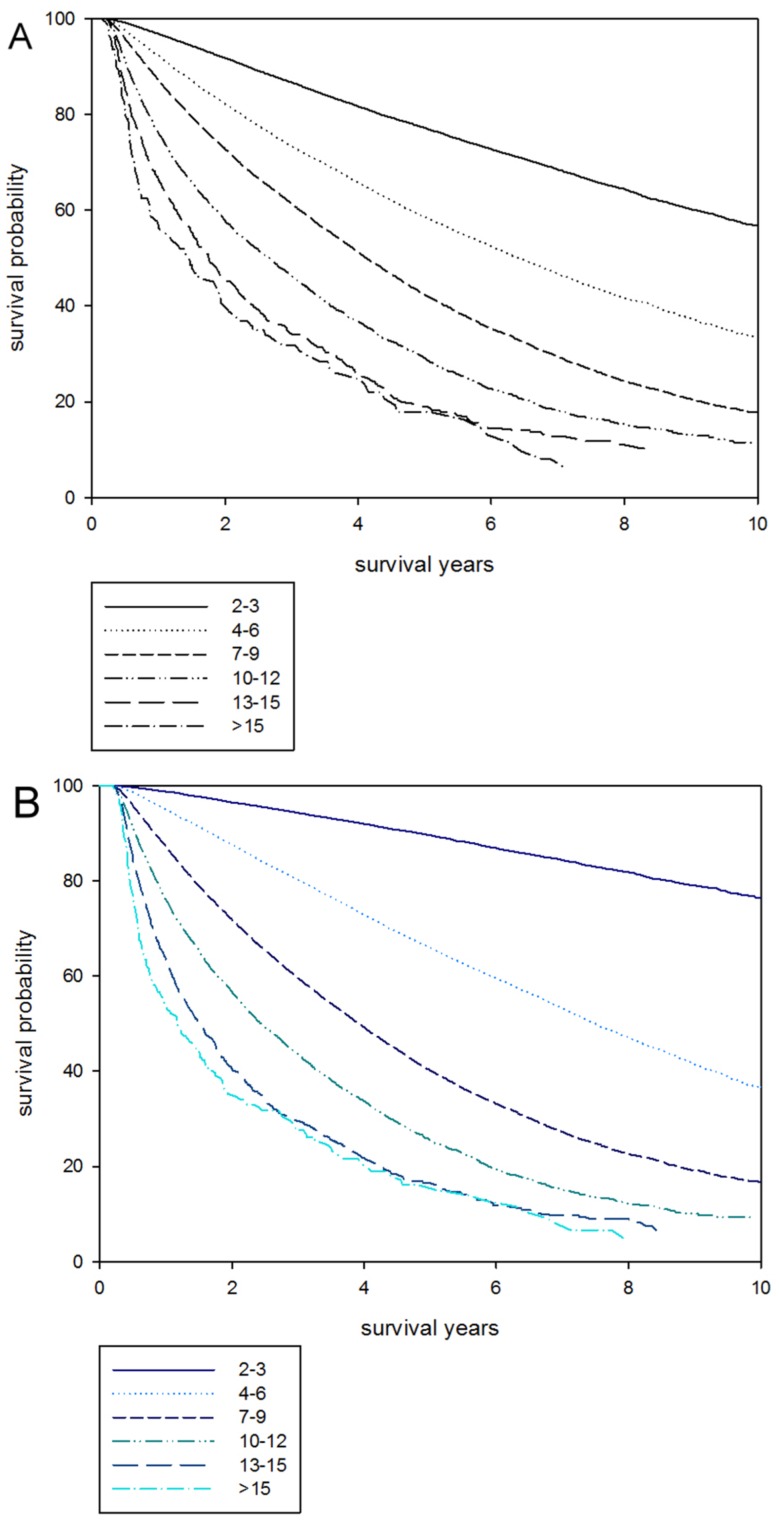
Survival curve stratified by Charlson comorbidity index (CCI) and age-weighted CCI. (A) Kaplan-Meier curves for 10 year survival by CCI score in incident patients. (B) Kaplan-Meier curves for 10 year survival by age-weighted CCI score. Survival is calculated beginning 90 days after starting dialysis. The survival rate of incident patients declined as CCI increased. A similar phenomenon is observed in incident patients in age-weighted CCI.

**Figure 3:**
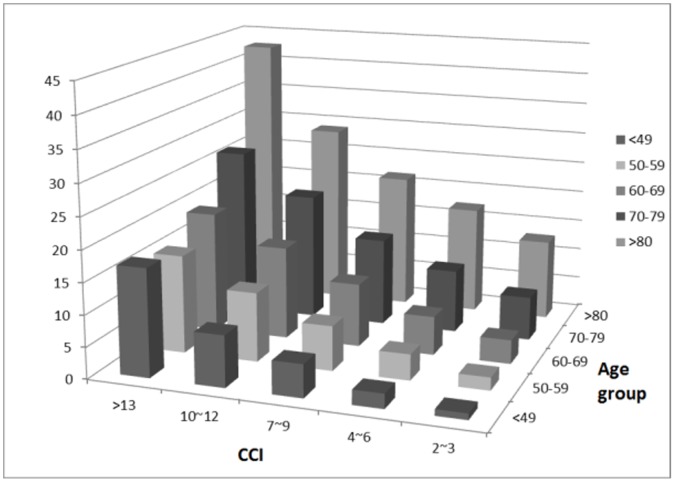
Increase of mortality with old age and high CCI in incident dialysis cohort. All the bars represent hazard ratio by age group and CCI.

The study population in the analyses was representative of the Taiwan dialysis population and enrolled 79645 incident patients with ESRD. Patient’s clinical characteristics and comorbid conditions are listed in Table 1. The patient’s mean age (±SD) was 60.96 (±13.92) years, 51.4% of patients were women, 51.2% were diabetic. After ranking subjects according to CCI scores, we categorized them into score quartiles with 10604, 30930, 24993, 9637, 2630, 851 numbers of patients in the six groups respectively. The distribution of CCI into six groups were 13.3% in index scores ≦ 3, 38.8% in index scores 4–6, 31.4% in index scores 7–9, 12.1% in index scores 10–12, 3.3% in index scores 13–15, and 1.1% in index scores >15. We made the same categorization in age-weighted CCI into six groups as listed in Table 1.Older patients were more likely to be in the higher comorbidity index (Figure 1). In cox proportional hazard models and stratifying by age, older patients had significantly higher mortality than younger patients: at age 30 to 39 years, the adjusted hazard ratio [aHR] was 1.20 (95% confidence interval [CI], 1.02–1.40), at age 40 to 49 years, the aHR was 1.74 (95% CI, 1.51–2.01), at age 50 to 59 years, the aHR was 2.71 (95% CI, 2.36–3.12), at age 60 to 69 years, the aHR was 4.11 (95% CI, 3.57–4.72), at age 70 to 79 years, the aHR was 6.27 (95% CI, 5.45–7.21), at age older than 80 years, the aHR was 10.40 (95% CI, 9.02–12.00), respectively. For every increased age of one year, the relative risk of death was 1.050 (95% CI 1.049–1.050, *p*-value <0.001) (data not shown). Males had higher risk of all-cause mortality compared to females significantly (aHR was 1.16, *p*-value <0.001). For CCI scores, mortality increased steadily with higher comorbidity both for unadjusted and for adjusted models. Compared to the lowest comorbidity group (reference group), fully adjusted models of CCI 4–6, CCI 7–9, CCI 10–12, CCI 13–15, CCI >15 showed hazard ratios for mortality of 1.91 (95% CI 1.81–2,02), 2.39 (95% CI 2.26–2.53), 2.42 (95% CI 2.28–2.57), 2.62 (95% CI 2.44–2.81), 2.78 (95% CI 2.53–3.05), respectively. For every increase of one point in the CCI score, the relative risk of death was 1.085 (95% CI 1.085–1.092, *p*-value <0.001) (data not shown). Mortality increased steadily with higher age or comorbidity both for unadjusted and for adjusted models. Figure 2A shows the survival curve for CCI stratified according to six comorbidity index groups. The same survival curve is illustrated in Figure 2B as age-weighted CCI. Death rates increased both with increasing age and increasing CCI score (Figure 3). Baseline mortality rates among elderly patients with CCI scores 2 to 3 points were higher than for younger patients. Among elderly patients with age more than 80 years and CCI more than 13 points, mortality rates were extremely higher than the CCI 10- to 12- point group (Figure 3). For all age groups, mortality rates increased in different CCI groups with the highest rates occurring in the oldest age groups.

**Table 1:** Basic demographics and characteristics of incident dialysis patients.

N = 79645	N	%
Age group		
18–19	136	0.2
20–29	1670	2.1
30–39	4386	5.5
40–49	11417	14.3
50–59	17697	22.2
60–69	21128	26.5
70–79	17751	22.3
≧80	5460	6.9
Gender		
Male	38683	48.6
Female	40962	51.4
Specific comorbidity		
Myocardial infarction	7873	9.9
Congestive heart failure	37168	46.7
Peripheral vascular disease	10,996	13.8
Cerebral vascular disease	29564	37.1
Dementia	5819	7.3
Chronic lung disease	31443	39.5
Rheumatological disorder	3614	4.5
Peptic ulcer disease	43637	54.8
Mild liver disease	21542	27.1
Diabetes with complications	40780	51.2
Paraplegia	3135	4.0
Neoplasia	15212	19.1
Moderate/severe liver disease	16559	20.8
Metastatic disease	2666	3.4
Human immunodeficiency virus	47	0.1
Conventional CCI		
≦3(n %)	10604	13.3
4–6	30930	38.8
7–9	24993	31.4
10–12	9637	12.1
13–15	2630	3.3
>15	851	1.1
Age-weighted-CCI		
≦3(n %)	5521	7.0
4–6	18330	23.1
7–9	27755	35.0
10–12	18921	23.8
13–15	6695	8.4
>15	2178	2.8

**Footnotes:** CCI, Charlson comorbidity index.

## Discussion

The present study used a nationally representative dialysis dataset to evaluate the ability of the Charlson comorbidity index to predict long-term survival in a large population of incident dialysis patients. Results suggest that the CCI is a good tool to assess comorbidity and predict survival in general dialysis population as previous studies validated [16], [22]–[24]. When CCI is compared directly to the Wright-Khan Index, Davies Index, and ICED, the areas under the Receiver Operator Characteristic (ROC) curve are 0.67, 0.68, 0.68, and 0.72 respectively. Furthermore, the CCI requires only 15 minutes (often less) to complete [25], and a useful comorbidity index should be a simplified utilitarian substitute for individual comorbid conditions.

Our study population demographics and clinical characteristics are not obviously different from a previously reported 1995–2002 Taiwan Renal Registry study [26] and other NHI published population based study [20]. The comorbid conditions are the same in other Taiwan dialysis cohort studies, as myocardial infarction [20], peripheral vascular disease [27], and diabetes mellitus [28]. In Taiwan, the national health insurance covers all expenses in the health-care system including hemodialysis and peritoneal dialysis. We calculated all the inpatient and outpatient diagnosis codes one year before dialysis to define the CCI, and expect missing comorbidity information to be rare and unlikely to influence the result. We demonstrated that old age, higher CCI, or higher age-weighted CCI posit lower survival rates (Table 2 and Figure 2). Age is still the strongest predictor of mortality in dialysis patients but is a better predictor when combined with CCI. The *p* for trend hazard ratio is higher in age-weighted CCI than age or CCI only. In the elderly patient, dramatically increased hazard ratio by CCI groups are found (Figure 3). Thus, we should pay more attention to incident elderly dialysis patients with higher comorbidity conditions, which cause higher mortality and lower survival rates. Though it is controversial to judge patients with ESRD as for dialysis no matter how many comorbid conditions exist -or with short survival expectation, and this involves ethical and medical problems, and quality of life and life expectancies should be considered in this elderly and high comorbidity group.

**Table 2:** The effect of age and CCI on the survival of dialysis patients by cox regression (univariate and multivariable).

Variable	CrudeHR	95% CI	*p*-value	Adjust HR	95% CI	*p*-value
Age group, years						
18–29	1(Ref.)	–		1(Ref.)	–	–
30–39	1.26	1.08–1.48	0.004	1.20	1.02–1.40	0.025
40–49	2.15	1.87–2.49	<0.001	1.74	1.51–2.01	<0.001
50–59	3.89	3.38–4.47	<0.001	2.71	2.36–3.12	<0.001
60–69	6.05	5.26–6.95	<0.001	4.11	3.57–4.72	<0.001
70–79	9.04	7.87–10.39	<0.001	6.27	5.45–7.21	<0.001
≧80	14.57	12.64–16.80	<0.001	10.40	9.02–12.00	<0.001
Sex						
Men	1.13	1.11–1.16	<0.001	1.16	1.13–1.182	<0.001
Women	1(Ref.)	–		1(Ref.)	–	
CCI						
≦3	1(Ref.)	–		1(Ref.)	–	
4–6	2.49	2.35–2.63	<0.001	1.91	1.81–2.02	<0.001
7–9	3.53	3.34–3.73	<0.001	2.39	2.26–2.53	<0.001
10–12	3.66	3.45–3.88	<0.001	2.42	2.28–2.57	<0.001
13–15	4.12	3.84–4.42	<0.001	2.62	2.44–2.81	<0.001
>15	4.42	4.02–4.86	<0.001	2.78	2.53–3.05	<0.001

**Footnotes:** CCI, Charlson comorbidity index; HR, Hazard ratio.

Our study has several limitations. First, laboratory data and measures of physical functioning are not available in the National Health Insurance Research Database. Though clinical parameters, such as predialysis systolic blood pressure [29], [30], calcium × phosphate product [31], hematocrit [32], novel inflammatory markers such as C-reactive protein or Il-6 [33], BMI [34], or nutritional status related to survival cannot be obtained from this data set, this study still showed an index of comorbidity is the strongest statistical predictor of mortality previously [24]. CCI was a better predictor than models containing age, diabetes, cardiovascular disease, or albumin [16]. Second, certain parameters that may have improved the performance of our study population, such as information for dialysis access, dialysis dose, modality, residual renal function, or other treatment factors during follow up were not included because of unavailability in the database. However, this was consonant with the study objective, to assess baseline risk factors for survival in an incident dialysis population. Third, we were unable to link some nonhospital deaths, thus we had to define the date of cancellation of health insurance as the date of death. Previous study had link data with the real death registration showings that, on average, most cancellation dates were within 1 week of the real death registration for dialysis patients [9]. Fourth, patients who received dialysis <90 days were excluded from this study because of the possibility of including patients with acute renal failure or terminal illness with renal failure patients. Our study also had several strengths. Firstly, claims data from universal medical coverage in Taiwan allow for identification of population samples free from selection bias and of sufficient size to document outcomes. Secondly, by using insurance records that consist of comorbidity information, we could unambiguously analyze comorbid conditions and survival rates in the incident dialysis patients.

### Conclusion

In conclusion, age and CCI, an index of overall comorbidity, assessed at the onset of dialysis, was the strong predictor of survival. Long-term survival rate was low in incident dialysis patients in the elderly and with higher comorbidity indexes. The simply CCI score should be emphasized in every incident dialysis patient to predict long term mortality and also evaluate his/her quality of life.

## Supporting Information

Table S1 **Deyo’s CCI (ICD-9CM).**
(DOCX)

Table S2 **Charlson comorbidity index (CCI) weight score.**
(DOCX)

Table S3 **Weighting for age.**
(DOCX)
