# Supplementary material for: Predicting Mortality of Incident Dialysis Patients in Taiwan - A Longitudinal Population-Based Study
Source: PLoS One. 2013 Apr 23;8(4):e61930. doi: 10.1371/journal.pone.0061930 (PMC3633990; doi:10.1371/journal.pone.0061930)
Supplement: Table S1 — Deyo’s CCI (ICD-9CM). (DOCX) [file pone.0061930.s001.docx]

Supplement table 1 Deyo’s CCI (ICD-9CM)

| Comorbid conditions | Deyo’s CCI (ICD-9-CM) |
| --- | --- |
| Myocardial infarction | 410.x, 412.x |
| Congestive heart failure | 428.x |
| Peripheral vascular disease | 443.9, 441.x, 785.4, V43.4 |
| Cerebrovascular disease | 430.x-438.x |
| Dementia | 290.x |
| Chronic pulmonary disease | 490.x-505.x, 506.4 |
| Rheumatic disease | 710.0, 710.1, 710.4, 714.0-714.2, 714.81, 725.x |
| Peptic ulcer disease | 531.x-534.x |
| Mild liver disease | 571.2, 571.4-571.6 |
| Moderate or severe liver disease | 456.0-456.21, 572.2-572.8 |
| Diabetes with complication | 250.x |
| Hemiplegia or paraplegia | 344.1, 342.x |
| Any malignancy | 140.x-172.x, 174.x-195.8, 200.x-208.x |
| Metastatic solid tumor | 196.x-199.1 |
| AIDS/HIV | 042.x-044.x |
