# Supplementary material for: Predicting Mortality of Incident Dialysis Patients in Taiwan - A Longitudinal Population-Based Study
Source: PLoS One. 2013 Apr 23;8(4):e61930. doi: 10.1371/journal.pone.0061930 (PMC3633990; doi:10.1371/journal.pone.0061930)
Supplement: Table S2 — Charlson comorbidity index (CCI) weight score. (DOCX) [file pone.0061930.s002.docx]

Supplement table 2 Charlson comorbidity index (CCI) weight score

| Comorbid conditions | Weighted score |
| --- | --- |
| Myocardial infarction | 1 |
| Congestive heart failure | 1 |
| Peripheral vascular disease | 1 |
| Cerebrovascular disease | 1 |
| Dementia | 1 |
| Chronic pulmonary disease | 1 |
| Rheumatic disease | 1 |
| Peptic ulcer disease | 1 |
| Mild liver disease | 1 |
| Moderate or severe liver disease | 3 |
| Diabetes with complication | 2 |
| Hemiplegia or paraplegia | 2 |
| Any malignancy | 2 |
| Metastatic solid tumor | 6 |
| AIDS/HIV | 6 |
