# Supplementary material for: Predicting Mortality of Incident Dialysis Patients in Taiwan - A Longitudinal Population-Based Study
Source: PLoS One. 2013 Apr 23;8(4):e61930. doi: 10.1371/journal.pone.0061930 (PMC3633990; doi:10.1371/journal.pone.0061930)
Supplement: Table S3 — Weighting for age. (DOCX) [file pone.0061930.s003.docx]

Supplement table 3 Weighting for age

| Age group | Weighted score |
| --- | --- |
| 0-49 years | 0 |
| 50-59 years | 1 |
| 60-69 years | 2 |
| 70-79 years | 3 |
| 80-89 years | 4 |
| 90-99 years | 5 |
